# Generation of high-titer viral preparations by concentration using successive rounds of ultracentrifugation

**DOI:** 10.1186/1479-5876-9-137

**Published:** 2011-08-17

**Authors:** Christine V Ichim, Richard A Wells

**Affiliations:** 1 Department of Medical Biophysics, University of Toronto, Toronto, ON M5G 2M9, Canada; 2 Discipline of Molecular and Cellular Biology, Sunnybrook Research Institute, Toronto, ON, M4N 3M5, Canada; 3 Department of Medicine, University of Toronto, Toronto, ON, M5G 2C4, Canada; 4 Department of Medical Oncology, Myelodysplastic Syndromes Program, Toronto Sunnybrook Regional Cancer Centre, Toronto, ON, M4N 3M5, Canada

## Abstract

**Background:**

Viral vectors provide a method of stably introducing exogenous DNA into cells that are not easily transfectable allowing for the ectopic expression or silencing of genes for therapeutic or experimental purposes. However, some cell types, in particular bone marrow cells, dendritic cells and neurons are difficult to transduce with viral vectors. Successful transduction of such cells requires preparation of highly concentrated viral stocks, which permit a high virus concentration and multiplicity of infection (MOI) during transduction. Pseudotyping with the vesicular stomatitis virus G (VSV-G) envelope protein is common practice for both lentiviral and retroviral vectors. The VSV-G glycoprotein adds physical stability to retroviral particles, allowing concentration of virus by high-speed ultracentrifugation. Here we describe a method report for concentration of virus from large volumes of culture supernatant by means of successive rounds of ultracentrifugation into the same ultracentrifuge tube.

**Method:**

Stable retrovirus producer cell lines were generated and large volumes of virus-containing supernatant were produced. We then tested the transduction ability of virus following varying rounds of concentration by ultra-centrifugation. In a second series of experiments lentivirus-containing supernatant was produced by transient transfection of 297T/17 cells and again we tested the transduction ability of virus following multiple rounds of ultra-centrifugation.

**Results:**

We report being able to centrifuge VSV-G coated retrovirus for as many as four rounds of ultracentrifugation while observing an additive increase in viral titer. Even after four rounds of ultracentrifugation we did not reach a plateau in viral titer relative to viral supernatant concentrated to indicate that we had reached the maximum tolerated centrifugation time, implying that it may be possible to centrifuge VSV-G coated retrovirus even further should it be necessary to achieve yet higher titers for specific applications. We further report that VSV-G coated lentiviral particles may also be concentrated by successive rounds of ultracentrifugation (in this case four rounds) with minimal loss of transduction efficiency.

**Conclusion:**

This method of concentrating virus has allowed us to generate virus of sufficient titers to transduce bone marrow cells with both retrovirus and lentivirus, including virus carrying shRNA constructs.

## Introduction

Viral vectors are commonly used to introduce exogenous genetic material in experimental systems, and have been used successfully in human gene therapy trials to treat patients with primary immunodeficiencies such as X-linked severe combined immunodeficiency (SCID)[1-3] and adenosine deaminase deficiency [1-3]. Suitable vectors frequently used in the laboratory and clinical setting include retroviral and lentiviral vectors. However, the ability to transduce difficult-to-infect cells such as primary hematopoietic cells, hematopoietic stem cells, and neuronal cells with these vectors is dependent on the ability to produce stocks of high viral titers [4,5].

Retro- and lentivirus is produced by transfecting producer cell lines with viral plasmids resulting in the production of virions that are released into the supernatant. Target cells may be transduced using the supernatant or alternatively by using supernatant that has been concentrated to increase the viral titer. Ultracentrifugation is one method that may be used to concentrate supernatant containing retroviral and lentiviral vectors that were pseudotyped with the G envelope glycoprotein of the vesicular stomatitis virus (VSV-G)[6-8]. In contrast to endogenous envelope proteins, VSV-G is a sturdy glycoprotein that can withstand the stresses of prolonged ultracentrifugation [7]. Furthermore, transduction with VSV-G coated virions occurs via membrane fusion [9] not by receptor-mediated uptake, thereby expanding the cellular tropism of the viral particles [10]

Nevertheless, even after concentration of virus, titers may still not be high enough for the successful transduction of difficult-to-infect cells such as primary bone marrow cells. This is especially relevant if the vector is not amenable to the production of high viral titers, as is often the case with shRNA vectors [11] One method of increasing the concentration of virus, in principle, would be to simply scale up and increase the volume of supernatant concentrated. However, the amount of viral supernatant concentrated in currently used protocols is limited by the capacity of the rotor tube, typically 30 mL. To yield a higher concentration of virus some protocols allow for a second round of ultracentrifugation [7] In these cases following one round of centrifugation, the supernatant is decanted into a waste container and the viral pellet remains in the bottom of the centrifuge tube. Another 30 mL of viral supernatant is added to the previously used ultracentrifuge tube that contains a viral pellet, and the tube is centrifuged a second time. Following this second round of centrifugation the supernatant is decanted and the virus is resuspended overnight.

Here we report that performing multiple successive rounds of ultracentrifugation of retrovirus pseudotyped using the VSV-G envelope protein additively increases the titer of viral preparations. We have observed that even after four successive rounds of ultracentrifugation (6 hours of centrifugation) the transduction efficiency of the retroviral particles remains uncompromised. We further observe that this protocol is suitable for concentrating shRNA lentiviral particles to a titer sufficient for transduction of bone marrow cells.

## Materials and methods

### Cell lines

The 293GPG packaging cell line [12] (kind gift from Dr. Richard Mulligan) was maintained in 293GPG medium (Dulbecco's Modified Eagles Medium (DMEM) with high glucose, L-glutamine and sodium pyruvate supplemented with 10% heat-inactivated FBS, G418, Tetracycline, puromycin and penicillin/streptomycin) as previously described [12]. NIH/3T3 and 293T/17 cells were obtained from ATCC and maintained in DMEM medium with 10% defined bovine calf serum (Hyclone Cat # SH30073.03) and penicillin/streptomycin.

### Creation of stable producer cell lines

293GPG cells were cultured in 15cm plates with 30 mL of 293GPG medium. 12 hours after removal of antibiotics, cells were transiently transfected with 25 μg of plasmid DNA using Lipofectamine 2000 (Invitrogen). In this study we used either the MMP retroviral vector [13,14] in which the cDNA for human NR2F6 (EAR-2) was subcloned upstream of an IRES-EGFP cassette [15], and also the MMP-EGFP control vector. Virus was collected on days 3 to 7, concentrated by centrifugation at 16,500 RPM for 90 minutes and used to transduce a second culture of 293GPG cells grown in 293GPG medium. Transduction of > 95% of cells was confirmed by flow cytometry. Stable producer cell lines were cultured in DMEM supplemented with G418, Tetracycline and puromycin.

### Generation of retrovirus

To produce virus, 293GPG cells were grown to confluence and culture media was replaced with DMEM supplemented with 10% heat-inactivated FBS and penicillin/streptomycin, free of tetracycline, puromycin and G418. Medium was changed every 24 hours. Viral supernatant was collected at 72, 96, 120, 144, and 168 hours. Supernatant was filtered through a 0.45 μm pore size polyethersulfone (PES) bottle-top filter (Nalgene, Thermo Fisher Scientific).

Supernatant from each time point was pooled and then ultracentrifuged.

### Ultracentrifugation

Beckman Ultra-Clear centrifuge tubes (Cat # 344058) were sterilized for 15 minutes by exposure to UV light in a biological safety cabinet. For each round of ultracentrifugation 30 mL of viral supernatant was centrifuged at 16500 rpm (RCF avg: 36026; RCF max: 49092) for 90 minutes at 4°C in a Beckman SW28 swinging bucket rotor lined with a Beckman Ultra-Clear centrifuge tube. Following centrifugation, medium was carefully decanted into a bleach-filled container. To obtain similar final volumes, for the final round of centrifugation as the medium was being decanted a P1000 pipette was used to remove the final drop of medium so that all tubes would be in similar final volumes. Centrifuge tubes where then either covered in parafilm and then stored at 4°C overnight in an up-right position, or returned to the rotor bucket and loaded with another 30 mL of viral supernatant for another round of ultracentrifugation under the conditions described above. Pellets were kept over-night at 4 degrees. The following day pellets were gently resuspended by pipetting 20 times using a P200 pipette, care being taken to minimize the creation of foam. Viral stocks from replicate centrifuge tubes were pooled and the pooled viral stock was titrated.

### Titration

Titers were determined by transducing 1 × 10^6 ^NIH/3T3 cells seeded in one well of a 6-well plate in 4 mL of medium containing 4 μg/mL of polybrene (Sigma). After 5 hours virus was washed off the NIH/3T3 cells and fresh medium was added. After 48 hours the number of cells expressing GFP was determined using flow cytometery and viral titers were calculated based on the proportion of transduced cells. Admittedly, this approach will only give an approximation of the true viral titre as we have not established that conditions ensure the transduction of only one viral particle per cell, neither have we controlled for the possibility that multiple particles could infect each cell.

### Transduction of bone marrow cells

12-week old C57Bl/6 mice were given 5 fluorouracil, 150 μg/g body mass, by intraperitoneal injection and humanely killed ninety-six hours later. Bone marrow was collected from femurs and tibiae and cultured in Iscove's Modified Dulbecco's Medium previously conditioned by culturing on OP-9 cells (T Nakano, Japan) for 72 hours, supplemented with fetal bovine serum (5%), c-Kit ligand conditioned medium (3%), Flt-3 ligand (30 ng/mL), TPO (30 ng/mL), IL-11 (30 ng/mL), Insulin (10 μg/mL), bovine serum albumin (0.5%), conditions that minimize differentiation but initiate cycling of long-term repopulating cells.

Following prestimulation, 2.0 × 10^6 ^cells were seeded per well of a 24 well plate in 400 μL of bone marrow culture medium, plus 4 μg/mL polybrene (Sigma) and 10 mM HEPES (Gibco-Invitrogen). 75-150 μL of retrovirus was added to the cells to give an MOI of what our method of titration estimated to be 100. One round of spin-infection was carried out by centrifugation at 3000 RPM on a Beckman GH 3.8 rotor for 45 minutes at room temperature. Forty-eight hours after retroviral transduction GFP-positive cells were assessed by flow cytometry.

### Generation of lentivirus

The packaging vectors pRSV Rev, pMD2.G (VSV-G) and pMDLg/pRRE, as well as the shRNA vector H1GIP (a kind gift from John Dick, University Health Network) were grown in STBL2 competent cells (Invitrogen, Carlsbad, CA) at 30 degrees. Plasmid DNA was extracted using the EndoFree Mega kit (Qiagen).

293T/17 cells were passaged 1:4 to 1:6 three times a week, before reaching 80% confluence. This passaging schedule was intended to maintain the cells at a density where they would be in a log state of proliferation, as well as to maintain them as individual cells (as opposed to cell aggregates) which would also increase transfection efficiency. Only early passages of the 293T/17 cells lines were used for the production of lentivirus, furthermore, batches of cells were not maintained in culture for more than a month. Care was taken to maintain 293T/17 cells endotoxin free.

293T/17 cells were transfected using the CalPhos Mammalian Transfection Kit (Clonetech, Palo Alto, CA) in 15 cm plates. Briefly, 12 × 10^6 ^cells were plated in a 15 cm dish the day prior to transfection. Two hours before transfection medium was aspirated and cells were fed 25 mL of fresh medium. Calcium Phosphate precipitates were prepared in 50 mL conical tubes in master mixes sufficient for transfecting 6 plates. Each plate received a solution containing 63.4 μg of DNA (28.26 μg of the H1 shRNA hairpin vector; 18.3 μg of pMDLg/pRRE; 9.86 μg of pMD2.G and 7.04 μg of pRSV Rev) and 229.4 μL of 2 M Calcium solution in a total volume of 3.7 mL. The transfection solution was incubated 20 minutes at room temperature and was then added drop wise to each plate. Plates were incubated overnight with transfection precipitate, and washed with PBS the next morning.

Lentiviral supernatent was collected after 24 and 48 hours. Supernatant was centrifuged in a table-top centrifuge for 10 minutes to remove debris and then pooled and filtered through a 0.45 μm pore size polyethersulfone (PES) bottle-top filter (Nalgene, Thermo Fisher Scientific). Ultracentrifugation was conducted as described above.

## Results

### Generation of stable 293GPG cell lines

**Figure 1:**
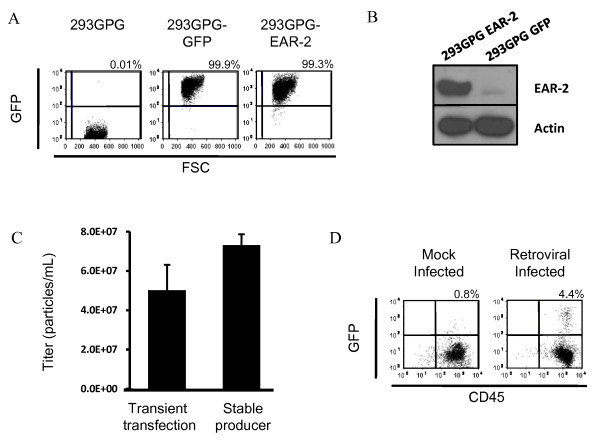
**Stable producer cell lines generated by transduction of 293GPG cells**. **A**. 293GPG stable producer cell lines for the GFP-empty vector control virus and the human EAR-2 -GFP virus are stable in expression of GFP. Flow cytometry performed after two months continuous culture shows GFP expression in > 99.5% of cells. **B**. 293GPG-EAR-2 cell lines were stable with respect to protein expression. Immunoblot analysis performed on transduced cells after two months continuous culture shows strong expression of EAR-2 protein. **C**. 293GPG stable producer cell lines were able to produce virus at titers significantly higher than those achieved by transient transfection. Virus was concentrated (one round). Error bars denote standard deviation. **D**. Transduction of bone marrow using virus produced from stable producer cell lines (1 round of ultracentrifugation) is not able to achieve high transduction rates in primary murine bone marrow cells.

293GPG cells were transformed into stable producer cell lines by transduction with retrovirus obtained from a previous round of viral production by transient transfection. We generated several polyclonal producer cell lines corresponding to a number of different viral constructs using the MMP backbone containing an IRES-GPF cassette. Polyclonal producer cells were stable over time in both expression of GFP (Figure 1A) and protein (Figure 1B). Although these lines produced virus at higher titres than those achieved by transient transfection of a suitable retroviral vector (MMP vector) (Figure 1C), we were not able to achieve high rates of transduction of bone marrow cells (Figure 1D), either using virus generated by transient transfection (data not shown) or from stable producer cell lines.

### Concentration of retrovirus using successive rounds of ultracentrifugation

**Figure 2:**
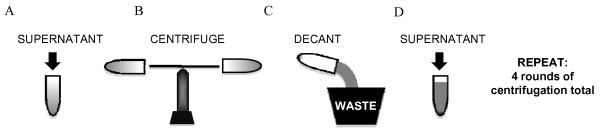
**Schematic of centrifugation protocol**.

While it is common protocol to concentrate VSV-G pseudotyped retrovirus by ultracentrifugation (Figure 2A-C), protocols recommend conducting a single round of centrifugation, with some giving the user the option of conducting a second round of centrifugation. Since our viral titres were not sufficiently high to transduce bone marrow cells we sought a method of increasing viral titres. We hypothesized that successive rounds of ultracentrifugation into the same centrifuge tube would allow the viral pellet to increase in size having an additive effect on viral titre.

The appeal of this protocol is that it is conceptually very simple: one fills a tube with virus containing medium (Figure 2A), the medium is centrifuged (Figure 2B), the virus is pelleted while the supernatant now devoid of virus is decanted into an appropriate biohazard waste receptacle (Figure 2C), the tube containing the pellet is then re-filled with more virus containing medium (Figure 2D) and they cycle is repeated for a total of four rounds of centrifugation. We chose four rounds arbitrarily for pragmatic reasons so that the centrifugation procedure may be finished in an 8-hour day.

**Figure 3:**
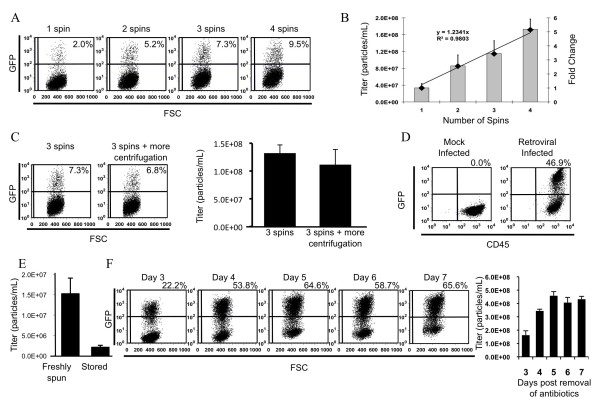
**Retrovirus coated with VSV-G may be concentrated using multiple rounds of centrifugation**. **A**. Assessment by flow cytometry of transduction by retrovirus following concentration using different numbers of rounds of centrifugation. 1 μL of retrovirus was added for each transduction. **B**. Titration of concentrated viral stocks. Bars denote the mean viral titer ± standard deviation. Diamonds represent the fold change in viral titer. The trendline shows a linear relationship between the fold change in viral titer and the number of rounds of centrifugation. **C**. Addition of an addition round of centrifugation without addition of unconcentrated supernatant does not result in a decrease in viral titre. **D**. Demonstration by flow cytometry of successful transduction of primary mouse bone marrow cells by retroviral particles concentrated using multiple rounds of centrifugation. **E**. Viral titers rapidly decrease following storage of virus at 4 degrees C for 7 days. **F**. Time course of viral titers obtained following four rounds of centrifugation of supernatant collected on the given day post-induction (removal of antibiotics/tetracycline). 5 μL of retrovirus was added for each titration.

To test whether we would be able to increase viral titres using sequential rounds of ultracentrifugation, medium from stable 293GPG producer cell lines that had been induced to produce virus by removal of antibiotics was concentrated by ultracentrifugation for a various numbers of rounds, and the concentrated stocks titred (Figure 3A and 3B). To reduce variation, supernatant used for these experiments taken was from a single batch of viral supernatant derived from pooling culture supernatant from numerous plates and filtered into the same bottle. Therefore, each experimental group was concentrated from supernatant with identical viral titers. Following the appropriate number of rounds of centrifugation centrifuge tubes were stored at 4 degrees. Upon titration we observed that viral titers indeed increased with each subsequent round of centrifugation (Figure 3A) and showed that this increase is additive, as demonstrated by the linear relationship in the fold change of viral titres (Figure 3B).

To test whether such long centrifugation periods had a detrimental effect on viral titres we compared the titres of two experimental groups that differed only in the amount of centrifugation they received (Figure 3C). Initially all tubes were subjected to three rounds of centrifugation, in which tubes were centrifuged, decanted and fresh viral containing medium added to the previous viral pellet. Following three rounds of centrifugation, half the tubes in the rotor were decanted and stored for at 4°C for titration the next day, while the other half of tubes were centrifuged an addition round (without decanting supernatant or addition of fresh viral medium), after which they too were decanted and stored at 4°C for titration the next day. Both groups hence contained the same quantity of virus, and differed only in the amount of centrifugation each received. We did not observe a significant difference in the titres between these two experimental groups (Figure 3C) suggesting a minimal effect of centrifugation on viral titres.

It is a common belief that centrifugation is able to pull down cellular debris, membrane fragments, and proteins from the virus containing medium. Conceivably, these putative byproducts might have a detrimental effect on any target cell, especially primary cells which are even more sensitive [16] Furthermore, it has been suggested that ultracentrifugation might concentrate factors inhibitory to viral transduction [17]. Given that the reason we wanted to increase viral titres was to transduce bone marrow cells, we tested the ability of our virus to transduce primary bone marrow cells from mice. We were able to achieve an outstanding transduction rate in primary bone marrow cells (Figure 3D).

Finally we were interested in studying some of the technical variables so as to achieve the highest possible titre using this method. Given that such a large quantity of viral supernatant is needed for four consecutive rounds of ultracentrifugation (30 mL × 6 rotors × 4 spins = 720 mLs), and given that it is possible to collect viral supernatant from the 293GPG producer cell line for up to day 7 after transient transfection, it is convenient to store the filtered supernatant at 4°C until the final day of collection, at which point concentration of the viral containing medium could commence. This approach is contingent upon the retrovirus remaining stable at 4°C. We directly tested whether storage of the viral supernatant at 4°C was detrimental to the transduction efficiency of the viral particles. To address this, a stock of concentrated viral supernatant was split two ways. One portion of the stock was titred immediately following resuspension of the viral pellet, while the remainder of that same viral stock was stored at 4°C for 7 days before the titer was determined. A striking decrease of nearly ten-fold in magnitude was observed in the viral titers from the stock that was stored at 4°C (Figure 3E). Based on these data, virus should be moved to long-term storage (-70°C) as soon as possible.

The observation that virus is not stable at 4°C (Figure 3E) suggests that it would be most efficient to design a scaled-up protocol in which sufficient culture supernatant could be collected to permit daily centrifugation, thereby minimizing the need for 4°C storage. This approach is contingent upon adequate concentrations of virus being present in the supernatants throughout the collection period; hence, we measured the variation in viral titres between days of collection in order to determine for how long culture supernatant collection from the producer cells can continue after withdrawal of tetracycline, G418 and puromycin. Supernatant collections began on day 3 and continued on to day 7. We observed that transduction efficiency varied little over this period, with the exception of day 3, on which transduction efficiency was consistently lower (Figure 3F). Notably, no decline in transduction efficiency was seen after day 3, suggesting that useful collection of supernatants might be extended beyond day 7.

### Concentration of lentivirus using successive rounds of ultracentrifugation

**Figure 4:**
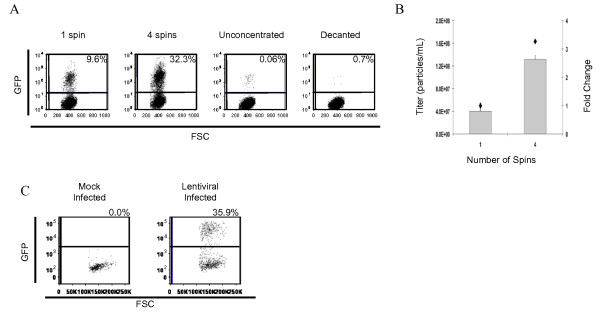
**Lentivirus coated with VSV-G may be concentrated using multiple rounds of centrifugation**. **A**. Titration of shRNA lentivirus following concentration by one round versus four rounds of centrifugation. Flow cytometry dot plots show the transduction rates following transduction with 5 μL of concentrated lentiviral stock, 50 μL of unconcentrated viral supernatant or 100 μL of supernatant that was decanted following a round of centrifugation. **B**. The increase in viral titres (bars) following successive rounds of centrifugation is additive as shown by the fold change relative to one round of centrifugation (diamonds). **C**. Lentiviral particles that are concentrated using multiple rounds of centrifugation are able to transduce primary mouse bone marrow cells.

The ability to generate high titre lentiviral stock capable of transducing bone marrow cells is of great experimental importance, and is a necessary step for the introduction of shRNA molecules into hematopoietic cells. Since lentiviral particles are often pseuodotyped with VSV-G, we investigated whether multiple rounds of centrifugation would have a similar additive effect on the titres of shRNA lentiviral particles pseudotyped with VSV-G, generated by calcium-phosphate transfection of four-plasmids into 293T/17 cells. Indeed, we observed that it was possible to increase the titre of a lentiviral stock in an additive manner by conducting four-rounds of ultracentrifugation (Figure 4A and 4B). Furthermore, we demonstrated that the lentiviral stock concentrated through four rounds of ultracentrifugation was able to transduce bone marrow cells (Figure 4C).

## Discussion

The introduction of exogenous genes into primary cells and difficult-to-transfect cells such as bone marrow requires the preparation of high titer viral stocks. 293GPG is a stable producer cell line that requires only the transfection of the viral backbone vector. We have generated stable specific producer lines by transducing 293GPG with virus generated by transient transfection. While this approach increased the ease with which virus is generated and the viral titres achievable, nevertheless, even after generation of stable producer cell lines concentrated viral supernatants still did not yield high transduction efficiencies in primary bone marrow cells. We sought to raise our viral titers further by increasing the quantity of viral supernatant that we concentrated. We determined that it is possible to increase the viral titers of the concentrated stock by conducting multiple rounds of ultracentrifugation. We observed a linear relationship between the number of rounds of ultracentrifugation and viral titer, suggesting that even after 4 rounds of ultracentrifugation the transduction efficiency of VSV-G coated retroviral particles were not adversely affected.

With the exception of our first day of collection we observed little fluctuation in transduction efficiency over time (Figure 3F). These results are in contrast with the results of Ory *et al *who showed that viral titers after transient transfection of 293GPG decreased several days post-transfection and illustrate an additional advantage of creating stable specific viral producer lines. It is important to note however that since the question we were addressing was "how many days can we collect for" our method of quantifying viral titres is not sufficiently stringent to address the question of weather there was a difference in the viral titres over time. Rather we designed the study to address merely whether in later time points we could attain a titre sufficient to transduce bone marrow cells based on our previous empirical observations. It is very well possible that the viral titres at later time points are much higher than those that we have measured, as it is possible that we have reached a plateau in the number of cells transduced and that the cells are being subjected to multiple retroviral integrations, We make no claims as to the absolute titres achievable, we only claim that virus can be produced from these stable producer cell lines until later time points (days 5-7 or perhaps longer) and that this virus is at least of sufficient titre for transduction of bone marrow cells.

Even short-term storage of viral supernatant at 4°C adversely affected the viral titer. Pragmatically, this suggests that in the execution of this protocol it is important to scale up the number of plates of 293GPG cells producing virus-containing supernatant, so that virus can be concentrated immediately after each collection. In our laboratory we have adopted a protocol that employs 25 plates which we grow with 30 mL of medium, and carry out centrifugations every day of medium collection.

The observation that it is possible to increase the titer of VSV-G coated retroviral particles simply by scaling up the amount of supernatant produced and then concentrating it by successive rounds of ultracentrifugation has broad applications. Although here we report concentrating VSV-G coated retrovirus from stable producer cell lines, we have previously used the strategy of successive rounds of ultracentrifugation to concentrate VSV-G coated retroviral particles generated by transient transfection. We have also shown that this principle can be applied to increase the titres of VSV-G coated shRNA lentiviral stock.

## Conclusions

In this study we found a reliable and robust method of increasing the concentration of VSV-G coated viral preparations by using multiple rounds of ultracentrifugation. This approach has appeal in that it is robust yet conceivably very simple. It is an easy technique which involves repetition and that does not require the mastery of yet another laboratory technique. It is a foolproof method of increasing viral titre that anybody can execute.

## Competing interests

The authors declare that they have no competing interests.

## Authors' contributions

CI and RW participated in the conception and design of the study. CI performed all experimental work. CI and RW wrote the manuscript. All authors read and approved the final manuscript.
